# Water Quality Changes Associated with Cassava Production: Case Study of White Volta Bain

**DOI:** 10.1016/j.heliyon.2016.e00149

**Published:** 2016-08-30

**Authors:** Alfred Awotwi, Michael Asare Bediako, Emmanuel Harris, Eric Kwabena Forkuo

**Affiliations:** a Department of Civil Engineering, Kwame Nkrumah University of Science and Technology, Ghana; b Department of Mathematics and Statistic, Takoradi Polytechnic, Ghana; c Department of Mathematics, Kwame Nkrumah University of Science and Technology, Ghana; d Department of Geomatic Engineering, Kwame Nkrumah University of Science and Technology, Ghana

**Keywords:** Cassava, Water quality, White Volta Basin, SWAT

## Abstract

The outcome reveal that as the land use in the catchment areas change from mixed agricultural to cassava cultivation, the simulated loads and concentrations of nitrogen species from cassava land-use scenario recorded reduction. The resultant concentrations of nitrate and nitrite for both current and future land-use scenarios are all below the daily limit suggested by the WHO, (World Health Organization). For the phosphate concentration, an increase of 4.21% was depicted under cassava land-use scenario. The results show that SWAT is a reliable water quality model, capable of simulating accurate information for developing environmental management plans.

## 1 Introduction

Water is important for human beings economic development and biological diversity hence the most indispensable of all natural resources. But a lot of countries have to face the challenge of rapidly growing water demands driven by an increased population and economic growth, linked to urbanization, industrialization and mechanization (Sundarakumar et al., 2012; Cohen, 2006), as well as the quality associated with water. The resulting water availability and its quality are among the most pervasive natural resources problems faced by water resources managers (Brels et al., 2008).

As water moves along the land surface, it carries the residues from the land. Surface runoff from different types of land use may be enriched with various kinds of contaminants. For example, runoff from agricultural lands may be enriched with nutrients and sediments. Likewise, runoff from highly developed urban areas mining sites may be enriched with rubber fragments, heavy metals, as well as sodium and sulphate. Evapotranspiration, infiltration, percolation and absorption of various types of vegetation cover and soil types can modify the land surface characteristics, water balance and hydrologic cycle (LeBlanc et al., 1997). Thereby altering the water quantity and water quality of receiving water bodies. It is therefore conceivable that there is a strong relationship between land-use types and the quantity and quality of water (Filoso et al., 2003; Brodie and Mitchell, 2005)

A research of Land use and topography as predictors of nutrient levels in a tropical catchment by Castillo (2010) revealed that highest nutrient concentrations are generated in agricultural lands. Also according to Van Der Velde et al. (2014) work on African crop yield reductions due to increasingly unbalanced nitrogen and phosphorus consumption showed that the agricultural practises in Africa produced higher amount of nitrogen and phosphorus. A study by Hoorman (2009) show that the quality of water can be linked to the type of crop. This relationship has also been established by the NRC (2008) with their work, Water implications of biofuels production in the United States, so investigating a particular crop in relation to water quality will help in unearthing the effect of economic crops.

Cassava the most important root crop in the tropics will have it production increased. This is due to the fact that is a major carbohydrate source for over 500 million people (Cock, 1985) and source of raw material for industries. As a result of good price and already existing market, farmers are converting their croplands to cassava farmlands. Although these kinds of landscape changes are unavoidable due to population increases and industrialisation, they will affect the hydrology and water quality in a watershed. This is because the types of ground cover and surface debris, evapotranspiration, infiltration, erosion, and sedimentation will be changed, thereby affecting not only the total quantities of pollutant loads but also the transport pathways of pollutant inputs (Tong and Chen, 2002; Zalidis et al., 2002). In order to safeguard our waterbodies from environmental degradation and to derive the maximum benefit from cassava production, it is imperative to have a better understanding of the interaction in the ecosystem, especially those relating to the water quality impacts on cassava production. Apart from future planning, development, and decision-making purposes, a reliable assessment tool is needed to predict the future water quality conditions under cassava scenario.

The main purpose of this study was to use a water quality model, soil and water analysis tool (SWAT), as a tool to assess the likely hydrologic and water-quality response of increasing cassava production, with particular reference to nutrient and sediment levels. Total nitrogen, total phosphorus and sediments yield were selected since (1) they are the basic water quality parameters and they can be used to assess the performance of water quality model, (2) they serve as indicators of eutrophication and nutrient enrichment encounter in any agricultural watersheds, and (3) they are only water quality parameters sampled by the centre for scientific and industrial research (CSIR), thereby providing the needed information for model calibration and validation

**Fig. 1:**
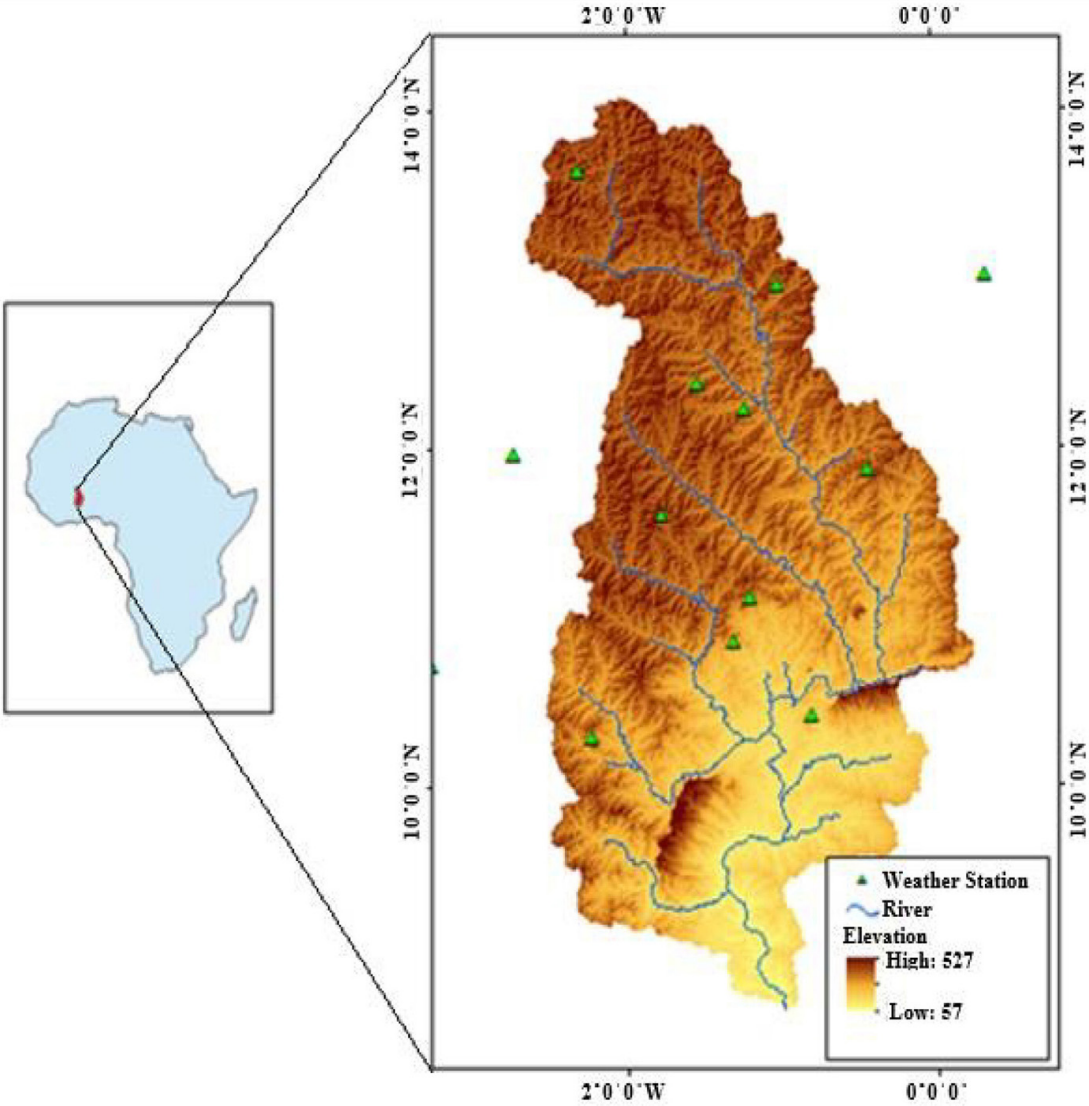
White Volta basin, the study area.

The White Volta River Basin in West Africa (Fig. 1) is selected as a case study, due to the conversion of croplands (especially yam, which has been the one of the major crop production in the area) to cassava production. The basin had experienced extensive cassava production in recent years, and can be attributed to (1) all year round planting period, (2) as raw material for starch and beer factories and (3) existence of already market. As the catchment becomes a high cassava production area, there would be considerable water resources consequences.

As a result of lack of information on the costs of pollution, national and local authorities in Ghana and Burkina Faso (location of the catchment) have not paid much attention to pollution control measures. In this study we modelled and quantified the effect of cassava production on water pollution. Our outcomes could help governmental bodies and non-governmental organisations enforce existing water pollution regulations. Our study could also provide useful information to authorities to manage water pollution and data for cost-benefit analyses.

## 2 Materials and methods

### 2.1 Study area

The White Volta catchment is located in the semi-arid and sub-humid zones of West Africa (Fig. 1), North of Lake Volta and lies between latitude 9°30′ N and 14°00′ N and longitude 2°30 W and 0°30 E (Awotwi et al., 2015). It covers mainly the northern part of Ghana and central part of Burkina Faso with an area of 106,000 km^2^. This catchment is one of the major catchments in Volta River System and contributes greatly to the livelihoods of tens of millions of people living in the basin (Ofosu et al., 2014; Amikuzuno, 2013).

The climate of the study area is being influenced by Inter-Tropical Convergence Zone (ITCZ) which controls the climate of West Africa, the location of the study area. The ITCZ moves across the Volta Basin in a complex manner resulting in uni-modal rainfall pattern in some areas and bi-modal rainfall pattern in other areas (Amisigo, 2005), with mean annual rainfall which varies between 900 mm from the northern part of the basin to about 1100 mm in the southern part. The temperature of the area decreases from northern part of the basin to the southern part with mean monthly temperature for the entire basin ranging between 28 °C in August and 39 °C in March (Awotwi et al., 2015).

In the White Volta catchment, most lands are used for agricultural activities. The food crops cultivated in this area are mostly rain-fed cultivation, including rice, millet, sorghum, maize; yam, cassava, groundnuts and beans. While in the dry season some farmers grow vegetables including tomatoes, pepper, okro, lettuce and cabbage. Free range animal grazing activities are practiced in the study area during the dry season, where livestock herdsmen move with their animals in search of water and feed in nearby communities (Allwaters Consult Limited, 2012). Soils found within the area are derived from granites, sandstones, alluvial materials, greenstone, andesite, schist and amphibolities (Obuobie, 2008). Specifically the soils are Fluvisol, Luvisols, Regosols, Vertisols, Lithosols, Planosols, Cambisols, Gleysols and Acrisols.

### 2.2 Description of SWAT Model

ArcSWAT, (an interface between ArcGIS and SWAT model), as a physically based, continuous, long-term, distributed-parameter hydrologic model, was used to simulate the water quality impacts under cassava. SWAT was chosen because it was developed specifically to simulate the effects of land management practices on flow, sediments, and agricultural chemical yields (Arnold et al., 1998). The major components of SWAT include hydrology, weather, erosion, plant growth, nutrients, pesticides, land management, and stream routing.

The model’s operations involve division of a catchment into sub-basins by overlaying land-use, soil map and Digital Elevation Model (DEM), and then further sub-divided into lumped units called Hydrologic Response Units (HRUs). HRUs are the percentages of the sub-watershed area. Soil water content, surface runoff, nutrient cycles, sediment yield, crop growth and management practices are simulated for each HRU and then aggregated for the subbasin by a weighted average.

Sediment yield in SWAT is estimated with the modified soil loss equation (MUSLE) developed by Williams and Berndt (1977). The MUSLE equation adapted for use in the model is:

$$Sed=11.8*{\left(Q_{surf}*q_{peak}*area_{hru}\right)}^{0.56}*K_{USLE}*C_{USLE}*P_{USLE}*LS_{USLE}$$

where Sed is the sediment yield, Q_surf_ is the surface runoff volume (mm water/ha), q_peak_ is the peak runoff rate (m^3^/s), area_hru_ is the area of the hydrologic unit area (HRU) in hectares, K_USLE_ is the USLE soil erodibility factor, C_USLE_ is the USLE cropping and management factor, P_USLE_ is the USLE conservation support practices factor, and LS_USLE_ is the USLE slope length and steepness factor. The Q_surf_ and q_peak_ are calculated every day precipitation occurs. If surface runoff occurs, then sediment yield is calculated for that day. Because crop growth affects Q_surf_ and q_peak_, C_USLE_ is also updated daily to reflect changes in the plant growth and land cover

Nutrients (nitrogen and phosphate) management and movement are simulated in SWAT using the modelling approach of GLEAMS. SWAT simulates the movement and transformations of nutrients between two minerals (ammonium and nitrate) and three organic (active, stable and fresh) soil pools.

Water yield is the total amount of water leaving the HRU and entering main channel during the time step. It is one of the important parameters that need to be estimated for sustainable management of water resources of the study area. It determines the concentration of pollutants being removed from the land area. The quantity of water yield affects the rate of soil erosion.

Water yield of a river catchment is estimated by the model using equation

WLYD=SURQ+LATQ+GWQ−TLOSS

where WYLD is the amount of water yield (mm H_2_O), SURQ is the surface runoff (mm H_2_O), LATQ is the lateral flow contribution to stream flow (mm H_2_O), GWQ is the groundwater contribution to stream flow (mm H_2_O) and TLOSS is the transmission losses (mm H_2_O) from tributary channels in the HRU via transmission through the bed.

### 2.3 Model input data sources

The data required in order to set up a SWAT model is classified into two, static and dynamics datasets. Static data includes DEM, soil and land-use whereas dynamics data comprises of climatic variables, discharge and water quality

### 2.4 Climate data

Daily temperature (maximum and minimum) and precipitation data from 14 weather stations (Fig. 1) located in the White Volta Basin were used as the climate input to run the SWAT model. Solar radiation, wind speed and relative humidity could not be used due to their unavailability. The data were obtained from the Ghana Meteorological Services Department and the Direction de la Météorologie Nationale, Burkina Faso, and covered the period 1995 to 2008.

*Soil map:* Soil from Soil and Terrain Database for West Africa was used to develop the soil map for the catchment. This was updated with FAO soils provided in the FAO soil database. Manually soil data were added into the SWAT soil database, soil parameters values were also added manually to White Volta catchment database. Eight different soil classes were identified in the study area: Luvisol soil is the dominant soil type in the basin followed by Regosols, Vertisol and Lithosols (Awotwi et al., 2015)

### 2.5 Land cover

**Fig. 2:**
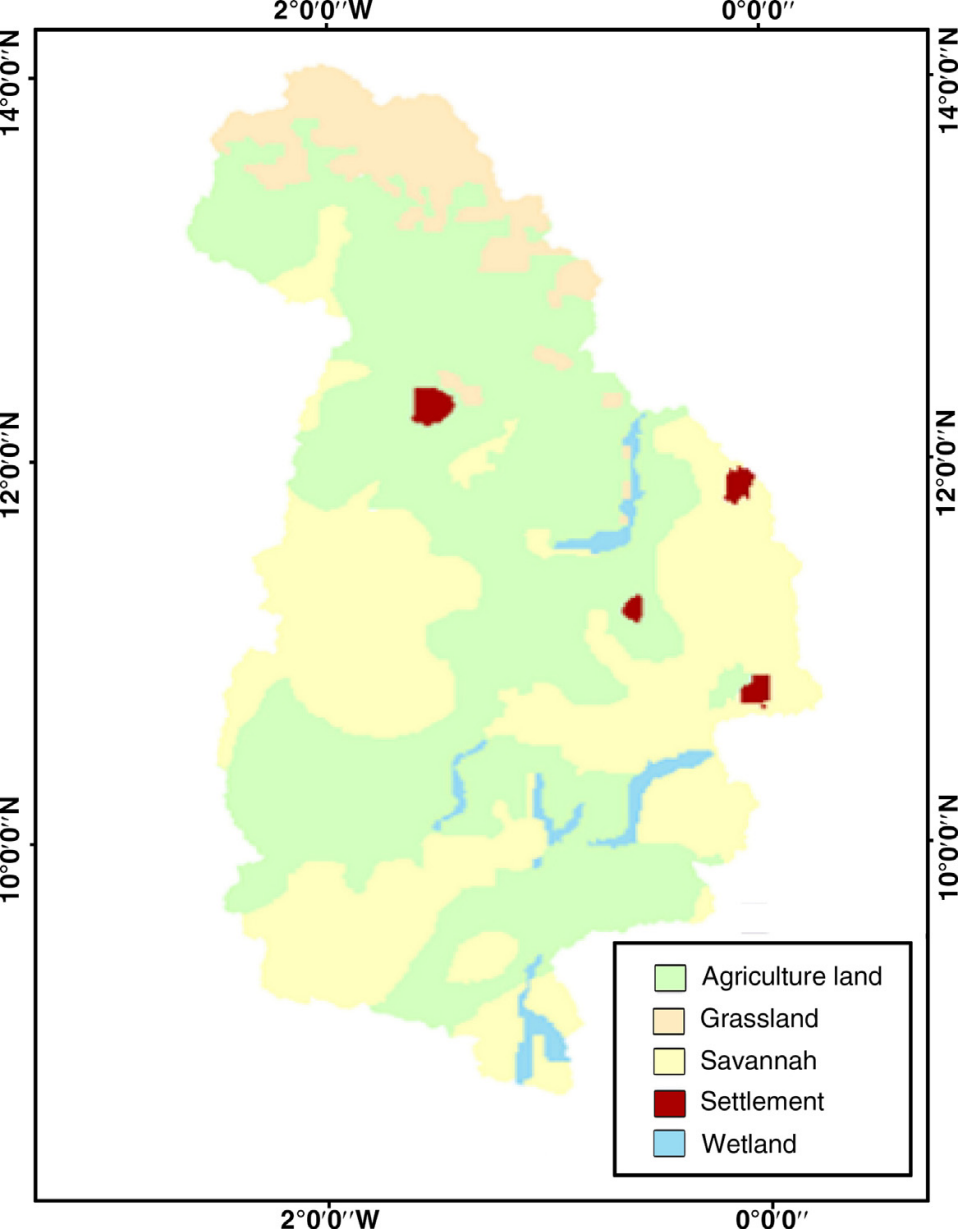
White Volta Catchment 2000s land use map.

One important factor that affects water balance components in a catchment is land cover. The LULC map, for the year 2000, used in this study was digitized from a map obtained from the Global Change and Hydrological Cycle website (GLOWA VOLTA-Maps, 2007). The map was georeferenced with ground truth data. The original legend was modified to make it suitable for SWAT modeling, of which the various parameters obtained from the CSIR, were added manually into the SWAT land cover database. With this modification of legends, four LULC, (land-use and land cover), classes were identified in the study area (Fig. 2), with the dominant class being cropland, savannah, grassland and urban.

*Digital Elevation Model (DEM):* A 90 m resolution DEM from the Shuttle Radar Topography Mission (SRTM) was used for the study (Fig. 1). The DEM was processed to remove sinks and other data errors.

*River discharge data:* Daily and monthly discharge data from Nawuni Guage Station which is located on the main White Volta River were used for calibrating and validating SWAT model. The discharge from Nawuni was used for the model due to the fact that it has the longest period data in the basin, the data were obtained from Ghana Hydrological Services (GHS).

*Water quality data*: Nitrate, nitrite and phosphate data was obtained from CSIR for March, July and October for 2006, 2007 and 2008 at Daboya and Pwalugu stations were used for calibration and validation respectively.

### 2.6 Statistics model evaluation

To assess how good the model is, procedures proposed by Haan et al. (1982) and further analyzed by Martinez-Rodriguez (1999), the Nash–Sutcliffe coefficient and coefficient of determination were chosen as the most suitable method for judging how good the model is during calibration and validation. For the calibration of the model to be accepted:

(1) the coefficient of determination is (R^2^) (Eq. (1)) should be greater than 0.50 (Santhi et al., 2001) and
(2) the Nash-Sutcliffe model Efficiency (NSE) (Eq. (2)) should be greater than 0.50.

R^2^ describes the proportion of the total variances in the observed data that can be explained by the model and ranges from 0 to 1. NSE is a measure of how well the plot of observed versus predicted values fit the 1: 1 line, and can theoretically vary from −∞ to 1, with 1 denoting a perfect model with respect to data agreement. Values between 0.0 and 1.0 are generally viewed as acceptable levels of performance, whereas values ≤0.0 indicates that the mean observed value is a better predictor than the simulated value, which indicates unacceptable performance (Moriasi et al., 2007) . NSE is a better representative measure for model goodness of fit (American Society of Civil Engineers (ASCE), 1993; Legates and McCabe, 1999).

(1) $$R^{2}=\left\{\frac{\sum_{i=1}^{n}\left(Q_{obs}-\bar{Q_{obs}}\right)\left(Q_{sim}-\bar{Q_{sim}}\right)}{{\left[\sum_{i=1}^{n}{\left(Q_{obs}-\bar{Q_{obs}}\right)}^{2}\right]}^{0,5}{\left[\sum_{i=1}^{n}{\left(Q_{sim}-\bar{Q_{sim}}\right)}^{2}\right]}^{0.5}}\right\}$$

(2) $$NSE=1-\frac{\sum_{i=1}^{n}{\left(Q_{sim}-Q_{obs}\right)}^{2}}{\sum_{i=1}^{n}{\left(Q_{obs}-\bar{Q_{obs}}\right)}^{2}}$$

Where Q_obs_ and Q_obs_ are observe value and mean of the observe values respectively while Q_sim_ and Q_sim_ are simulated value and mean of the simulations values. n is the number of observation.

### 2.7 Model calibration and validation

In order to better reflect the existing conditions, the SWAT model parameters were calibrated until there was a satisfactory agreement between the simulated flowrate and the observed flow data. The calibration of the model was done manually by adjusting parameters throughout the catchment, taking one parameter at a time and calculating the efficiency of the model before changing to the next value of the same or another parameter. The parameters were manipulated within their ranges.

The calibration was performed on daily streamflow data from Nawuni station, concentrations of nitrate, nitrite and phosphate from Daboya station. The parameters used for the model calibration area shown in Table 1. Daily streamflow data from January 1, 1995, to December 31, 2004 was used for calibration with the year 1995–1999 data used to warm up the model. March, July and October water quality data; nitrate, nitrite and phosphate concentrations, for 2006, 2007 and 2008 were also used to perform the calibration. The validation, assessing the ability of the model to predict observations for time periods different from the calibration period, was performed using daily discharge data of January 1, 2005, to December 31, 2008. While that of water quality was done using March, July and October nitrate, nitrite and phosphate concentrations, for 2006, 2007 and 2008 at Pwalugu station. Model performance was evaluated using the Nash-Sutcliffe coefficient (NSE) [Eq. (2)] and the coefficient of determination (R^2^) [Eq. (1)]

## 3 Results and discussion

### 3.1 Calibration and Validation

**Fig. 3:**
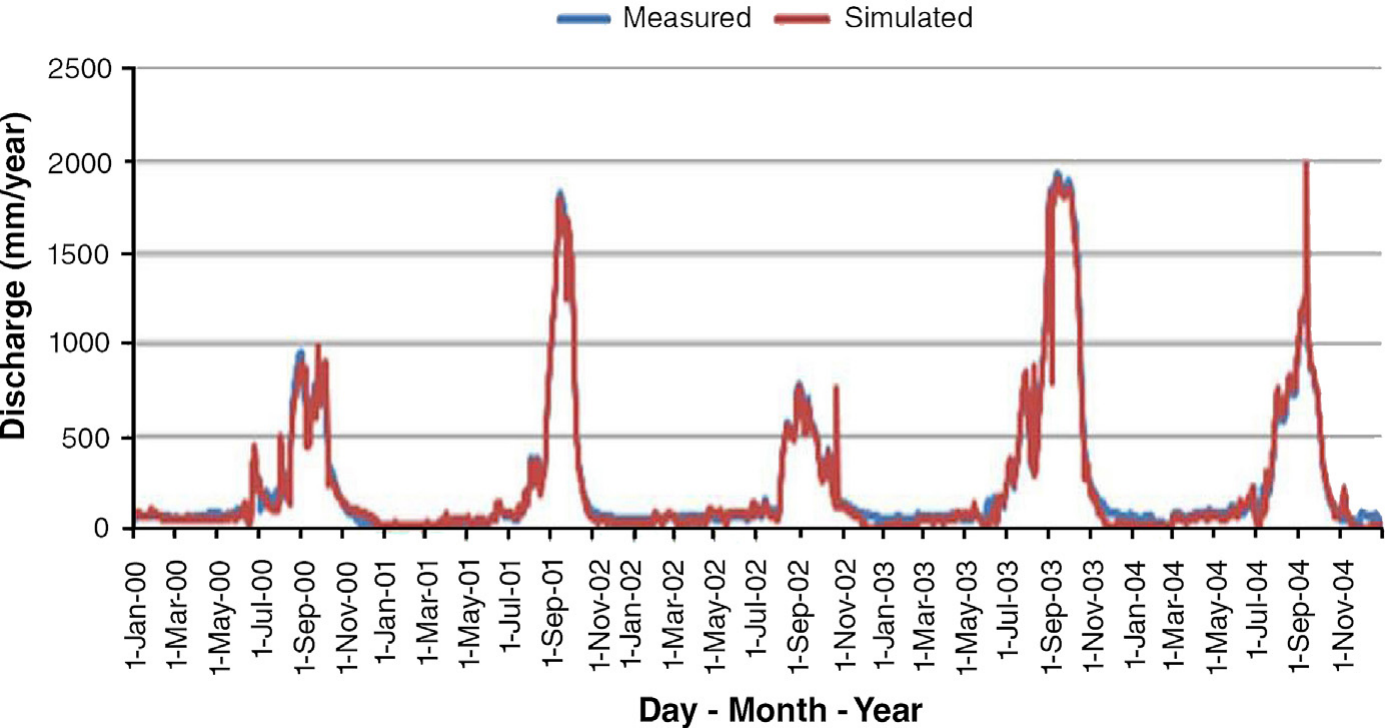
Calibration result of daily simulated and observed flow.

According to results of calibration, Fig. 3, over-estimation and under-estimation of the discharge was observed at the peak flow and low flow period respectively. This over-estimated may be attributed to lack of accounting for water withdrawal for irrigation data and climate change effect, which would also affect the model. While that of under-estimated may be due to more than one aquifer contributing to baseflow which can be handled in the SWAT model.

**Fig. 4:**
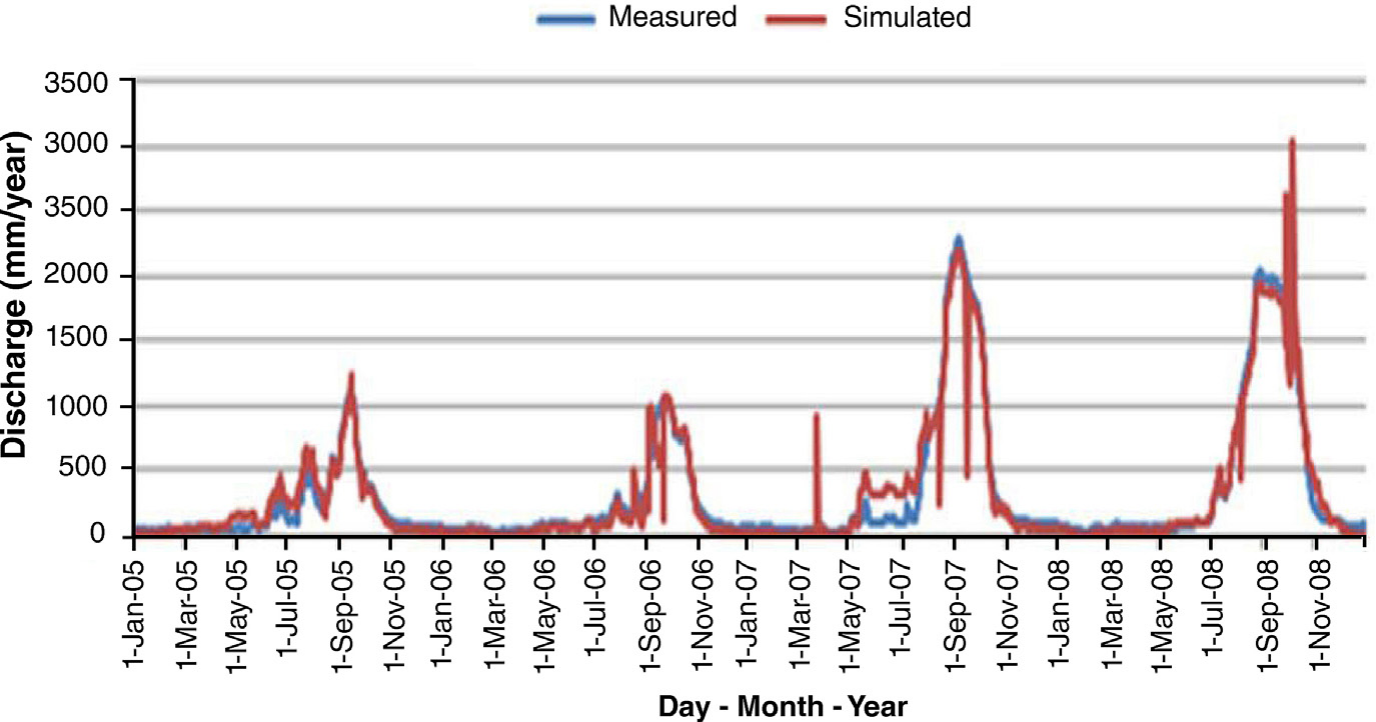
Validation result of daily simulated and observed flow.

Based on the statistical values of R^2^ of 0.83 and NSE of 0.78, (Table 2), the simulated discharge during calibration have “very good” agreement with observed discharge according to Moriasi et al. (2007) and also revealed by Fig. 3. Fig. 4, results of the validation, portrayed a good correlation between the observed flow and the simulated flow, indicated by R^2^ of 0.74 and NSE of 0.81.

**Fig. 5:**
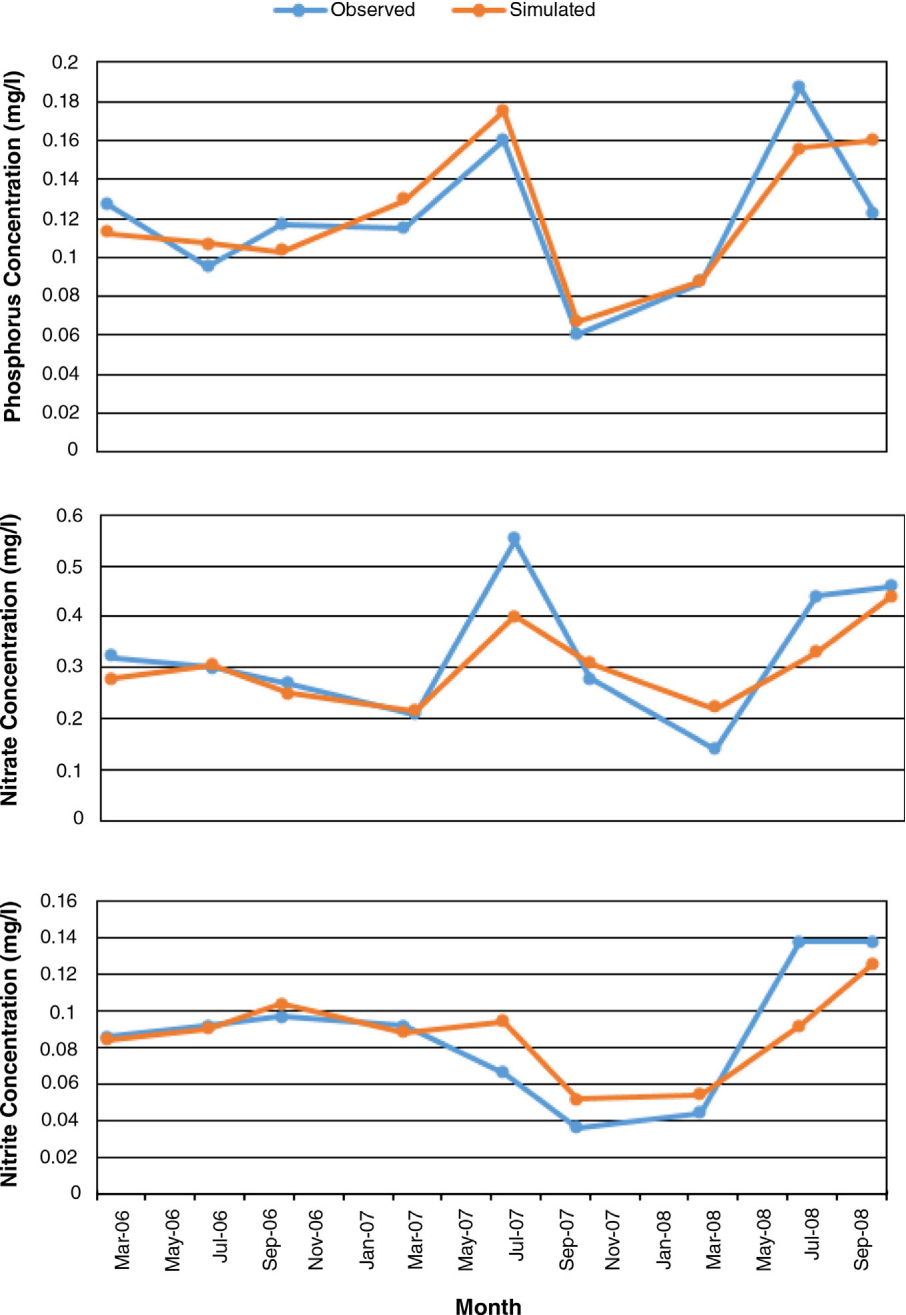
Calibration result of water quality components at Daboya station.

In simulating the water quality parameters, modelling was confined to nitrate, nitrite and phosphate. This is due to the fact that correlation analysis showed that these three variables had strong correlations with landuse types (Tong and Chen, 2002). Besides, there is a lack of available historical monitoring date on other water quality parameters for the basin. Three water quality calibration (nitrate, nitrite and phosphate) was performed based on the data from Daboya station. Table 1 presents all the parameters that were adjusted for water quality calibration. As in the hydrologic modelling, the simulated results for each water quality parameter were compared to the observed values from Daboya station and calibration was performed as described earlier. According to the statistical values (R2 and NSE) from Table 2 and graph, Fig. 5, reveal that the calibration were “very good” for Daboya station according to the ranges given by Moriasi et al. (2007). Usually good calibration of hydrology results in better sediment calibration which, in turn, leads to better phosphorus and nitrogen calibration.

Model performance measures at the calibration station, Fig. 5, shows that SWAT slightly overestimated phosphate. This may be due to (i) the influence of dams for irrigation along the White Volta River, (ii) a difference from the normal White Volta River fate and transport phenomena because of stream characteristics (3) lack of information about use of fertilizer, so its generalization does not accurately reflect actual practices in the basin. Also nitrate and nitrite were underestimated, this might be as a result of nonpoint agricultural sources to river nitrogen loads and decrease infiltration and leaching rate, which ultimately carry nitrogen loads to ground and surface waters.

**Fig. 6:**
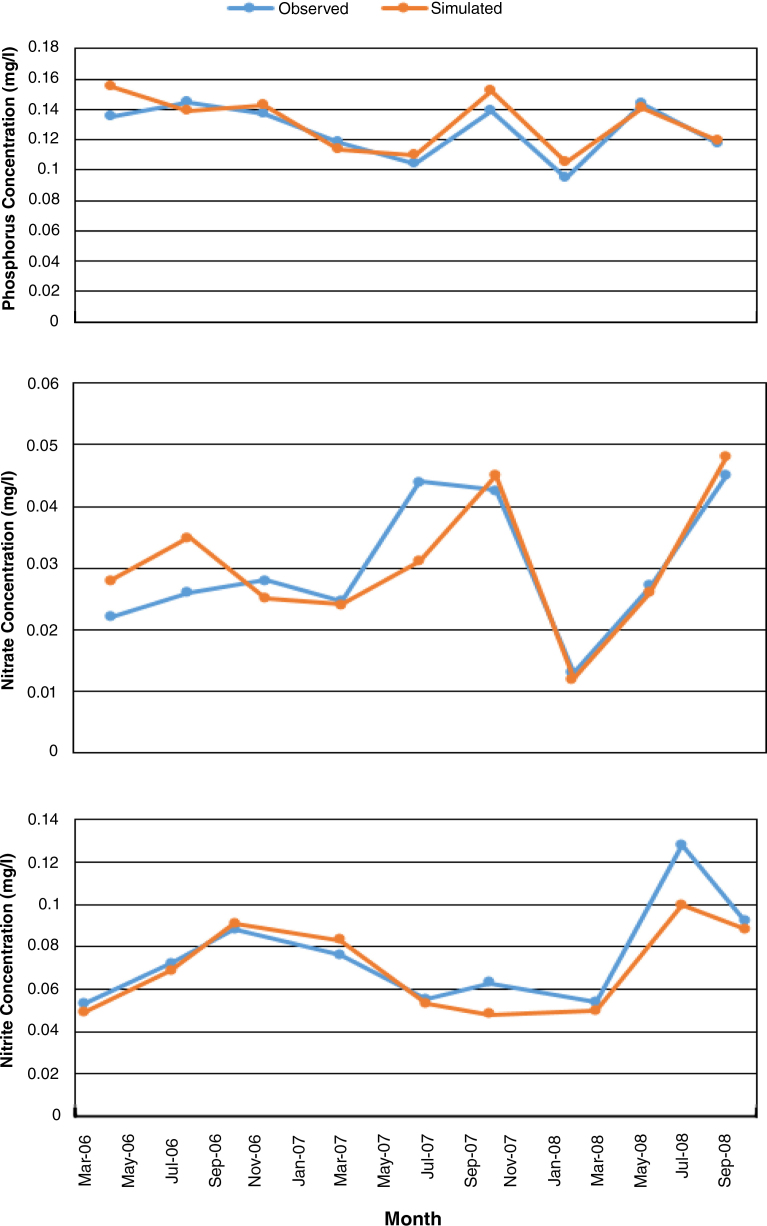
Validation result of water quality components at Pwalugu station.

The same picture of the estimation was observed during validation at Pwalugu station (Fig. 6), where phosphate was overestimated, and nitrate and nitrite underestimated. The statistical results (Table 2) reveal that the simulated data is very good agreement with observed data.

### 3.2 Impacts of land use change on water yield

Water yield is one of the important parameters to be estimated for efficient water management and planning of the case study area. Effects of land use change on water yield are derived from comparisons between the model results from current land use and future land use scenarios. These results of change in annual water yield according to various land use scenarios are summarized in Table 3. The model results indicate that the annual water yield under the cassava land-use scenario is about 96 mm H_2_O, which is 77.45% increase of the current land-use scenario (Table 3). This increment is as a result of decrease in infiltration caused by the lumping of the agriculture land to cassava land use because the soil types remain unchanged. This is due to the fact that in the SCS equation, the curve number, is dependent on the combination of land use and soil types. A lower curve number means a decrease in surface runoff as the land use/soil type combination is less resistant to infiltration (Jain et al., 2006). As the curve number increases, the land use/soil type combination present is more resistant to infiltration, and the amount of surface runoff increases.

### 3.3 Water quality with land use variations

From Table 3 the sediment yield increased by 92.61%, from 0.4941metric ton/ha to 0.9517 metric ton/ha, with the introduction of cassava. The increase of the yield may be due to the fact that cassava provides less cover for the soil surface (Lorsirirat and Maita, 2006) and decrease infiltration rate. Sediment yield tends to increase as water infiltration decreases.

The increase in sedimentation will have negative effect on the ecosystem; impeding the drainage network, destroys biodiversity, reduces storage capacity and water depth in lake and reservoirs, causing flooding. Most importantly, the potential for pollutants to be present increases due to increase in quantity of sediment. This is due to the fact that pollutants are bound to the sediment (Tsai et al., 2003; Jartun et al., 2008). Suspended sediment is important to characterize water quality since nutrients, such as phosphorus and nitrogen, can be transported in association with fine sediments.

**Fig. 7:**
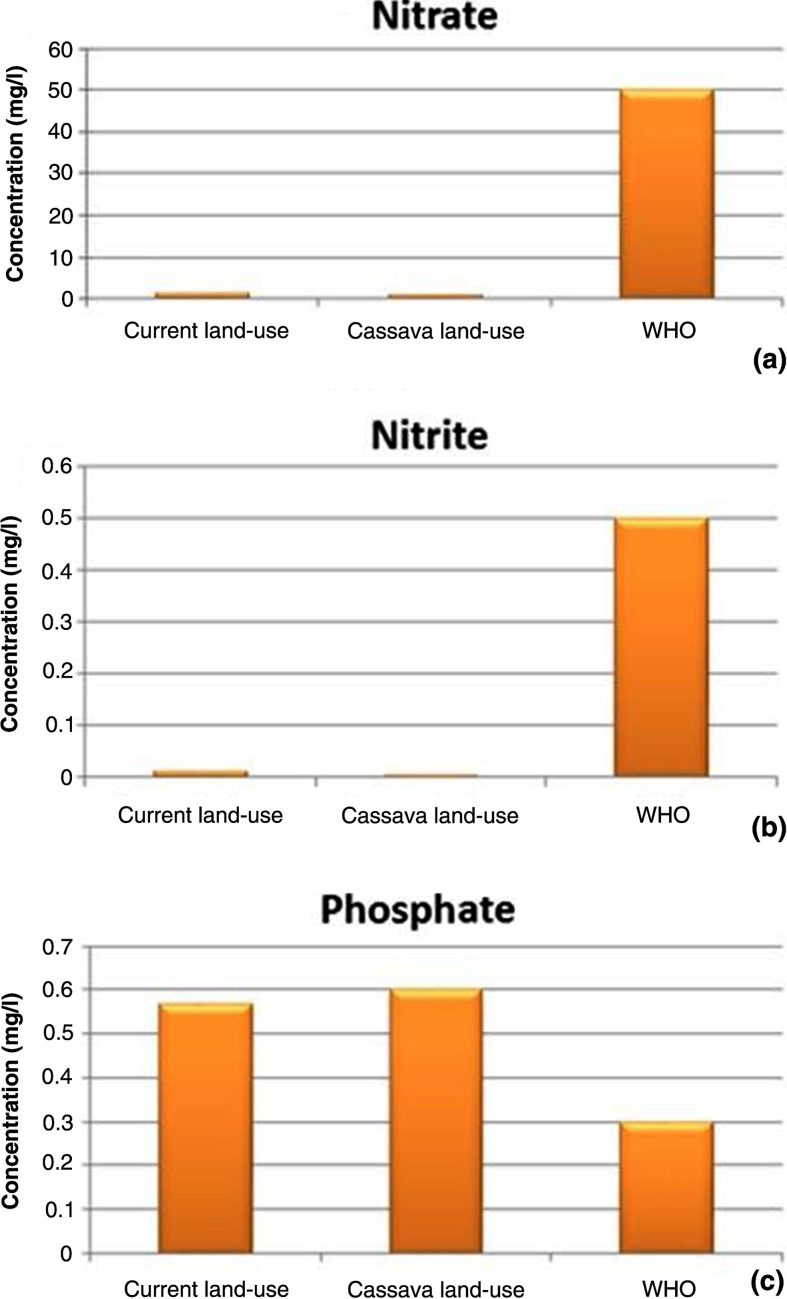
Concentration level of (a) nitrate, (b) nitrite and (c) phosphate, for current land use, cassava land use and WHO.

Table 3 depicts the comparison of the annual load and the mean daily concentration of all the nitrogen species modelled under the current and cassava land-use scenarios. The study shows all the nitrogen species recorded reduction in both the annual load and the mean daily concentration. The load and concentration of nitrate results show reduction of 7.88% and 28.40% respectively in the cassava land-use scenario. The concentrations of nitrate from current and cassava land-use scenarios were far below the WHO (1993) guideline value of 50 mg/l (Fig. 7(a)), and therefore the river is not polluted with nitrate. With the nitrite, the cassava land-use scenario recorded decrease of 9.64% and 53.04% in load and concentration respectively. The new nitrite concentration of 0.0058 mg/l is far below the WHO suggested limit of 0.50 mg/l (Fig. 7(b)). From our estimates, nitrogen decreases with decrease in infiltration. This outcome may be due to ability of the cassava to extract large amount of nitrogen. Similar results were reported by Agbaje and Akinlosotu (2004) and Adjei-Nsiah (2010), they reported high absorption of nitrogen by cassava. This may attributed to the root system increases the water uptake so as maintains requisite osmotic pressure (Jaleel et al., 2008) which is the medium of transporting nutrients. The decreased in nitrogen can be due to dilution, denitrification, and uptake by plants (Hubbard and Sheridan, 1989).

According to Table 3, the cassava land-use scenario, depicted increase of 30.17% in the total annual load of phosphate. With the mean daily phosphate concentration, an increase of 5.70%, from 0.5687 mg*/*l to 0.6011 mg*/*l was recorded. This may be due to the high content of phosphorus in the storage roots (Tewe, 2004; Odoemenem and Otanwa, 2011) as well as the increased in sediment yield. Since total phosphorus flux in rivers can be in association with suspended sediment (Ongley, 1996). The concentrations of current and cassava land-use scenarios are all above the World Health Organisation (WHO) guideline (Fig. 7(c)) value of 0.3 mg/l (WHO, 1993), hence, showing that there is the possibility of increase in eutrophication problem under the cassava land-use scenario due to an overabundance of phosphate. The increased in the phosphate concentration is as a result of decrease in infiltration rate.

The ratio of total nitrogen concentration to total phosphorus concentration was assessed to determine healthiness of the White Volta River. The ratio of total nitrogen concentration to total phosphorus concentration reduced by 26.15%; from 3.39 to 2.50. Both current and cassava land-use ratios are below the recommended ratio of 16:1 for healthy water bodies (Horne and Goldman, 1994). The ratio of 2.5 under the cassava scenario portrays that there is either lack of nitrogen or more of phosphate. Since the average daily concentrations of the nitrogen species do not exceed WHO’s suggested level (Fig. 7), but that of phosphorus does, it is likely that the eutrophication of the river system will be as a result of overabundance of phosphorus rather than nitrogen.

## 4 Conclusion

Cassava for biomass production can be an economically beneficially practice. However, water pollution may occur. In this study, the impacts of the cassava land use scenario on the watershed were determined successfully. The assessment of water quality responses on cassava land use scenario in the White Volta Basin indicated that cassava have effects on water yields and water quality components.

From the outcome of this study all nitrogen species experienced reduction in both average daily concentration and annual load under cassava land-use scenario. This may be due to the ability of the cassava to absorb the nitrogen since increased in sediment and surface runoff should have resulted in increased nitrogen species. The reduction is below the WHO suggested limit of 50 mg/l and 0.50 mg/l for nitrate and nitrite respectively, and is welcomed in light of health related issues with nitrogen The total phosphate concentration increased by 73.81%, from 0.2787 mg*/*l to 0. 4844 mg*/*l. this increment is a not a good result since is above the WHO guideline value of 0.3 mg/l.

These simulated effects of land use to cassava production clearly indicate a situation of depleting water quality in the catchment. In White Volta Catchment, we recommend that policies addressing this problem should be formulated both at the local and national level. Parallel to this, an intensive information and education campaign on the consequences of land use conversion and ways of rehabilitating the catchment should likewise be done.

The results obtained from the SWAT model indicate the capability of the model to adequately represent a West African watershed. The model is also capable of adequately assessing effects of different land cover conditions. The study has provided useful baseline information on the water quality of the White Volta Catchment for the management of the ecosystem as well as the ecosystem of the entire Volta basin, especially given the current Volta Basin Project, which is to support sustainable water resource management in the riparian countries of the Volta basin in West Africa.

## Declarations

### Author contribution statement

Alfred Awotwi: Conceived and designed the experiments; Performed the experiments; Analyzed and interpreted the data; Contributed reagents, materials, analysis tools or data; Wrote the paper.

Michael A. Bediako, Emmanuel Harris: Performed the experiments; Analyzed and interpreted the data; Contributed reagents, materials, analysis tools or data.

Eric K Forkuo: Analyzed and interpreted the data; Contributed reagents, materials, analysis tools or data.

### Funding statement

This research did not receive any specific grant from funding agencies in the public, commercial, or not-for-profit sectors.

### Competing interest statement

The authors declare no conflict of interest.

### Additional information

No additional information is available for this paper.

## Figures and Tables

**Table 1:** Parameter Selection and final value for Calibration of SWAT.

Hydrology			
Parameters	Default	Final value	Definition
CN2	−999	61	Initial SCS CN II value
ESCO	0.95	0.8	Soil evaporation compensation factor
ALFA_BF	0.048	0.95	Base flow alpha factor (days
SOL_AWC	−999	0.32	Available water capacity
GWQMN	1000	350	Threshold depth of water in the shallow aquifer
GW_REVAP	0.02	0.1	Groundwater “revap” coefficient
CH_K2	0	15.4	Effective hydraulic conductivity in main channel

Phosphorus			
Parameters	Default	Final value	Definition
PHOSKD	175	104	Phosphorus soil partitioning coefficient
PPERCO	10	5.3	Phosphorus percolation coefficient
PSP	0.4	0.56	Phosphorus sorption coefficient
SOL_ORGP	0	45	Initial organic P concentration in the soil

Nitrogen			
Parameters	Default	Final value	Definition
NPERCO	0.2	0.62	Nitrogen percolation coefficient.
SOL_NO3	0	58	Initial NO3 concentration in the soil
BIOMIX	0.2	0.68	Biological mixing efficie
SOL_ORGN	0	73	Initial organic N concentration

**Table 2:** Statistical results of the calibration and validation of the SWAT model.

	Station	Nawuni	Daboya			Pwalugu		
		Discharge	NO_2_	NO_3_	PO_4_	NO_2_	NO_3_	PO_4_
Calibration	R^2^	0.83	0.87	0.93	0.91			
NSE	0.78	0.68	0.68	0.81			
Validation	R^2^	0.74				0.76	0.69	0.7
NSE	0.81				0.77	0.84	0.89

**Table 3:** Comparison of water quality parameters under current and cassava land-use scenarios.

	Current	Future	Percentage Change
WAYD (mm/yr)	53.9217	95.6864	77.4543

Load	Current	Future	Percentage Change
SEYD (metric tons/ha)	0.4941	0.9517	92.6128
ORGN (kg N/ha)	1.6435	1.5297	−8.4704
LONO_2_ (kg N/ha)	0.0415	0.0375	−9.6386
LONH_4_ (kg N/ha)	0.0844	0.0568	−32.7014
LONO_3_ (kg N/ha)	0.0406	0.0374	−7.8818
LOPO_4_ (kg P/ha)	0.5313	0.6916	30.1713

Concentration	Current	Future	Percentage Change
CONCNO_2_ (mg/l)	0.0135	0.0058	−57.037
CONCNO_3_ (mg/l)	1.3416	0.9606	−28.3989
CONCNH_4_ (mg/l)	0.0895	0.0695	−22.3463
CONCORN (mg/l)	0.4828	0.4686	−2.9411
CONCPO4 (mg/l)	0.5687	0.6011	5.6972

WAYD = Water yield, SYLD = Sediment yield, ORGN = Organic nitrogen yield, LONO_2_ = Nitrite load, LONH_4_ = ammonia load, LONO_3_ = Nitrate load, LOPO_4_ = phosphorus load, CONCNO_2_ = Nitrite concentration, CONCNO_3_ = Nitrate concentration, CONCNH_4_ = ammonia concentration, CONCORN = Organic nitrogen concentration, CONCPO_4_ = Total phosphorus concentration.

## References

[bib0005] Adjei-Nsiah S. (2010). Yield and nitrogen accumulation in five cassava varieties and their subsequent effects on soil chemical properties in the forest/savanna transitional agroecological zone of Ghana. Journal of Soil Science and Environmental Management.

[bib0010] Agbaje G.O., Akinlosotu T.A. (2004). Influence of NPK fertilizer on tuber yield of early and late-planted cassava in a forest alfisol of south-western Nigeria. African Journal of Biotechnology.

[bib0015] Allwaters Consult Limited, 2012. Hydrological study of the Black Volta Basin – Upper West Region water audit section. Diagnostic study of the Black Volta Basin in Ghana final report. Available from: http://www.gwiwestafrica.org/sites/default/files/6_gh8_hydrological_study_of_the_black_volta_basin.pdf (accessed 01 June 2015).

[bib0020] Amikuzuno, J., 2013. Climate Change Implications for Smallholder Agriculture and Adaptation in the White Volta Bas in of the Upper East Region of Ghana. 4th International Conference of the African Association of Agricultural Economists. Available from: http://ageconsearch.umn.edu/bitstream/160478/2/Climate%20Change%20Impact%20on%20Smallholder%20Farmers%20in.pdf (accessed 23 May 2015).

[bib0025] Amisigo, B.A., 2005. Modelling riverflow in the Volta Basin of West Africa: A data driven framework. PhD Thesis. Ecology and Development Series No. 34. ZEF Bonn. Cuvillier Verlag, Göttingen. Available from: http://repository.tudelft.nl/islandora/object/uuid:dcda25cb-d872-4e6d-bca2-1bb0401c8d78?collection=research (accessed 12 November 2015).

[bib0030] Arnold J.G., Srinivasan R., Muttiah R.S., Williams J.R. (1998). Large area hydrologic modeling and assessment–Part 1: Model development. Journal of the American Water Resources Association.

[bib0035] ASCE (1993). Criteria for evaluation of watershed models. Journal of Irrigation and Drainage Engineering.

[bib0040] Awotwi A., Yeboah F., Kumi M. (2015). Assessing the impact of land cover changes on water balance components of White Volta Basin in West Africa. Water and Environment Journal.

[bib0045] Brodie J.E., Mitchell A.W. (2005). Nutrients in Australian tropical rivers: changes with agricultural development and implications for receiving environments. Mar. Freshwater Res..

[bib0050] Brels S., Coates D., Loures F. (2008). Transboundary water resources management: the role of international watercourse agreements in implementation of the CBD. CBD Technical Series no. 40, 48 pages. Secretariat of the Convention on Biological Diversity.

[bib0055] Castillo M.M. (2010). Land use and topography as predictors of nutrient levels in a tropical catchment. Limnologica.

[bib0060] Cock J.H. (1985). Cassava: New Potential for a Neglected Crop.

[bib0065] Cohen B. (2006). Urbanization in developing countries: Current trends, future projections, and key challenges for sustainability. Technology in Society.

[bib0070] Filoso S., Martinelli L.A., Williams M.R., Lara L.B., Krusche A., Victoria Ballester M., Victoria R., de Camargo P.B. (2003). Land use and nitrogen export in the Piracicaba River basin, Southeast Brazil. Biogeochemistry.

[bib0075] GLOWA VOLTA-Maps (2007). Land Cover in the Volta Basin Area. http://www.zef.de/fileadmin/template/Glowa/img/Landcover_Satellite_01.jpg.

[bib0080] Haan C.T., Johnson H.P., Brakensiek D.L. (1982). Hydrologic Modeling of Small Watersheds.

[bib0085] Hoorman J.J. (2009). Using Cover Crops to Improve Soil and Water Quality. http://www.mccc.msu.edu/states/Ohio/OH_CoverCrops_to_Improve_Soi%26Water_Quality.pdf.

[bib0090] Horne A.J., Goldman C.R. (1994). Limnology.

[bib0095] Hubbard R.K., Sheridan J.M. (1989). Nitrate Movement to Groundwater in the South-eastern Coastal Plain. Journal of Soil and Water Conservation.

[bib0100] Jain M.K., Mishra S.K., Singh V.P. (2006). Evaluation of AMC‐dependent SCS CN‐based models using watershed characteristics. Water Resources Mgmt..

[bib0105] Jaleel C.A., Gopi R., Sankar B., Gomathinayagam M., Panneerselvam R. (2008). Differential responses in water use efficiency in two varieties of Catharanthus roseus under drought stress. Comp. Rend. Biol..

[bib0110] Jartun M., Ottesen R.T., Steinnes E., Volden T. (2008). Runoff of particle bound pollutants from urban impervious surfaces studied by analysis of sediments from stormwater traps. Science of the Total Environment.

[bib0115] LeBlanc A., Papadopoulos M., Bélair C., Chu W., Crosato M., Powell J., Goodyer C.G. (1997). Processing of Amyloid Precursor Protein in human primary neuron and astrocyte cultures. Journal of Neurochemistry.

[bib0120] Legates D.R., McCabe G.J. (1999). Evaluating the use of ‘goodness-of-fit’ measures in hydrologic and hydro climatic model validation. Water Resources Research.

[bib0125] Lorsirirat K., Maita H. (2006). Soil Erosion Problems in Northeast Thailand: A Case Study from the View of Agricultural Development in a Rural Community Near Khon Kaen. Disaster Mitigation of Debris Flows, Slope Failures and Landslides.

[bib0130] Martinez-Rodriguez J.G. (1999). Sensitivity Analysis Across Scales and Watershed Discretization Schemes Using ARDBSN Hydrological Model and GIS. PhD Dissertation Submitted to the School of Renewable Natural Resources.

[bib0135] Moriasi D.N., Arnold J.G., Van Liew M.W., Bingner R.L., Harmel R.D., Veith T.L. (2007). Model evaluation guidelines for systematic quantification of accuracy in watershed simulations. American Society of Agricultural and Biological Engineers.

[bib0140] NRC (National Research Council) (2008). Water implications of biofuels production in the United States. http://dels.nas.edu/resources/static-assets/materials-based-on-reports/reports-in-brief/biofuels_brief_final.pdf.

[bib0145] Obuobie E. (2008). Estimation of groundwater recharge in the context of future climate change in the White Volta River Basin. Doctoral thesis.

[bib0150] Odoemenem I.U., Otanwa L.B. (2011). Economic Analysis of Cassava Production in Benue State, Nigeria. Current Research Journal of Social Sciences.

[bib0155] Ofosu E.A., Zaag P., van der Giesen, van de N., Odai S.N., Amanor R. (2014). Analysis of Upscaling of Irrigation Development in the White Volta sub-Basin. JENRM.

[bib0160] Ongley E.D. (1996). Control of Water Pollution from Agriculture. FAO Irrigation and Drainage Paper No. 55.

[bib0165] Santhi C., Arnold J.G., Williams J.R., Dugas W.A., Srinivasan R., Hauck L.M. (2001). Validation of the SWAT model on a large river basin with point and nonpoint sources. J. Am. Water Resources Assoc..

[bib0170] Sundarakumar K., Harika M., Begum S.K.A., Yamini S., Balakrishn K. (2012). Land use and land cover change detection and urban sprawl analysis of Vijayawada city using multitemporal landsat data International. Journal of Engineering Science and Technology.

[bib0175] Tewe O.O. (2004). Cassava for livestock feeding in Sub-Saharan Africa. ftp://ftp.fao.org/docrep/fao/007/j1255e/j1255e00.pdf.

[bib0180] Tong S.T.Y., Chen W. (2002). Modeling the relationship between land use and surface water quality. Journal of Environmental Management.

[bib0185] Tsai L.J., Yu K.C., Ho S.T., Chang J.S., Wu T.S. (2003). Correlation of Particle Sizes and Metals Speciation in River Sediment. Diffuse Pollution Conference.

[bib0190] Van Der Velde M., Folberth C., Balkovic J., Ciais P., Fritz S., Janssens I., Obersteiner M., See L., Skalsky R., Xiong W., Penuelas J. (2014). African crop yield reductions due to increasingly unbalanced Nitrogen and Phosphorus consumption. Global Change Biology.

[bib0195] WHO (1993). Guidelines for drinking water quality. Volume l Recommendations.

[bib0200] Williams J.R., Berndt H.D. (1977). Sediment yield prediction based on watershed hydrology. Transactions of ASAE.

[bib0205] Zalidis G., Stamatiadis S., Takavakoglou V., Eskridge K., Misopolinos N. (2002). Impacts of agricultural practices on soil and water quality in the Mediterranean region and proposed assessment methodolog. Agriculture. Ecosystems and Environment.

